# Dehydration of Lipid Membranes Drives Redistribution of Cholesterol Between Lateral Domains

**DOI:** 10.1021/acs.jpclett.4c00332

**Published:** 2024-04-18

**Authors:** Hanna Orlikowska-Rzeznik, Emilia Krok, Maria Domanska, Piotr Setny, Anna Lągowska, Madhurima Chattopadhyay, Lukasz Piatkowski

**Affiliations:** 1 Faculty of Materials Engineering and Technical Physics, Poznan University of Technology, 60-965 Poznan, Poland; 2 Biomolecular Modelling Group, Centre of New Technologies, University of Warsaw, 02-097 Warsaw, Poland

## Abstract

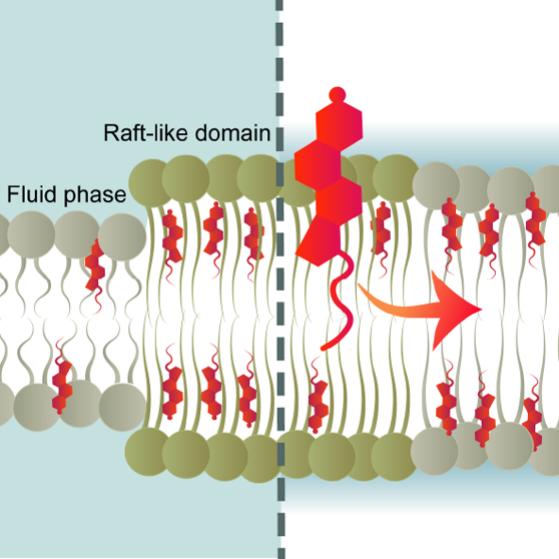

Cholesterol-rich lipid rafts are found to facilitate membrane fusion, central to processes like viral entry, fertilization, and neurotransmitter release. While the fusion process involves local, transient membrane dehydration, the impact of reduced hydration on cholesterol’s structural organization in biological membranes remains unclear. Here, we employ confocal fluorescence microscopy and atomistic molecular dynamics simulations to investigate cholesterol behavior in phase-separated lipid bilayers under controlled hydration. We unveiled that dehydration prompts cholesterol release from raft-like domains into the surrounding fluid phase. Unsaturated phospholipids undergo more significant dehydration-induced structural changes and lose more hydrogen bonds with water than sphingomyelin. The results suggest that cholesterol redistribution is driven by the equalization of biophysical properties between phases and the need to satisfy lipid hydrogen bonds. This underscores the role of cholesterol–phospholipid–water interplay in governing cholesterol affinity for a specific lipid type, providing a new perspective on the regulatory role of cell membrane heterogeneity during membrane fusion.

Cell membranes extend well beyond their role as mere physical barriers, serving to maintain cellular integrity. Hydrated lipid bilayer assemblies, abundant in proteins, sustain a far-from-equilibrium state crucial for the energetic and dynamic nature of cells. This enables cells to execute essential biochemical processes for life, adaptation, and response to their environment.

Expanding upon the fundamental fluid mosaic model,^1^ our understanding of cell membrane architecture has evolved. The current perspective describes it as an intricate, asymmetric lipid bilayer structure with nonrandomly distributed components.^2^ Cell membranes exhibit lateral heterogeneity, marked by transient nanodomains, known as relatively ordered “lipid rafts”, which are enriched in cholesterol and sphingolipids.^3^ These rafts are surrounded by a fluid phase, characterized by the abundance of unsaturated phospholipids.^3^ The prevailing consensus is that the molecular structure of the lipid rafts closely resembles that of the liquid ordered (L_o_) phase, while the fluid regions are effectively modeled as the liquid disordered (L_d_) phase, coexisting in biomimetic cell membranes composed of ternary lipid mixtures (saturated and unsaturated phospholipids and cholesterol).^4^

Lipid rafts have been suggested to be pivotal in various cellular processes, including signal transduction,^5^ membrane protein trafficking,^6^ and host–pathogen interactions.^7^ A growing body of evidence emphasizes the role of cholesterol-rich lipid rafts in the entry of various viruses into target cells, such as acute respiratory syndrome coronavirus 2 (SARS-CoV-2),^8^ dengue,^9^ ebola,^10^ influenza A,^11^ and human immunodeficiency virus (HIV).^12−16^ Most of these viruses utilize the endocytic route for cell entry, whereas HIV enters T lymphocytes through membrane fusion at the cell surface.^17^ Notably, the HIV fusion peptide exhibits structural changes dependent on the target membrane cholesterol level, adopting mostly an α-helical conformation in the low cholesterol condition but shifting toward a β-sheet secondary structure with increasing cholesterol content.^18^ This observation raises the intriguing possibility that membrane regions differing in cholesterol content may be sequentially engaged in the fusion process, or alternatively, that the cholesterol level is actively regulated at the fusion site. Further studies using the mimics of HIV envelope and target T cell membrane revealed that high content of cholesterol, and particularly the phase separation into fluid L_d_ and raft-like L_o_ domains, facilitates the membrane fusion process.^14,15^ Intriguingly, the L_o_–L_d_ phase boundaries have been unveiled to serve as preferential binding sites for the HIV fusion peptide.^14,15^

Membrane fusion is a central phenomenon not only in viral entry, which leads to the pathological condition, but also in a whole palette of biological processes such as fertilization^19^ or neurotransmitter release by exocytosis,^20^ which have also been shown to be affected by membrane cholesterol content. Despite the evolutionary and structural diversity of the proteins involved, these processes share a common pathway characterized by a series of distinct intermediates. The initial loose protein-mediated membrane contact is followed by tight apposition of the membranes, leading to local, transient membrane dehydration.^21^

In a quest to gain a more detailed picture of the role of cholesterol and lipid rafts in the membrane fusion process at the stage of dehydration, we pose the questions: What is the specific organization of cholesterol in the membrane fusion dehydration intermediate, and why might the boundaries of raft-like domains promote the fusion process? To address these queries, we employed confocal fluorescence microscopy and molecular dynamics (MD) simulations. We focused on the lateral distribution of cholesterol between coexisting L_o_ and L_d_ phases in planar solid-supported lipid bilayers (SLBs) under varying hydration conditions, which were precisely controlled by slow and sequential changes in the relative humidity (RH) of the membrane environment.

We investigated the membranes composed of an equimolar mixture of the unsaturated lipid 1,2-dimyristoleoyl-glycero-3-phosphocholine (14:1 PC), egg sphingomyelin (SM), and cholesterol (Chol). The 14:1 PC:SM:Chol (1:1:1) SLBs exhibit microscopic phase separation at room temperature into L_o_ domains and the surrounding L_d_ phase. The L_o_ domains consist of sphingomyelin and a high fraction of cholesterol and are characterized by a high degree of ordering of fatty acid chains, and consequently dense molecular packing and decreased intramolecular dynamics. In contrast, the surrounding L_d_ phase is composed of the unsaturated lipid 14:1 PC and a lower cholesterol fraction. The L_d_ phase is less tightly packed and exhibits greater fluidity due to steric constraints arising from the unsaturated acyl chains.

The lateral distribution of cholesterol was monitored as a function of membrane hydration state using a fluorescently labeled cholesterol derivative, modified at the terminus of the alkyl backbone, TopFluor-cholesterol, often referred to as BODIPY-cholesterol.^22^ For control purposes, the L_d_ phase was labeled with the unsaturated lipid 1,2-dioleoyl-*sn*-glycero-3-phosphoethanolamine (DOPE) labeled at the headgroup with the Atto 633 fluorescent probe. Exemplary fluorescence microscopy images of a double-labeled SLB at four membrane hydration states (bulk hydration, 85%, 60%, and 38% RH) are depicted in Figure 1.

**Figure 1:**
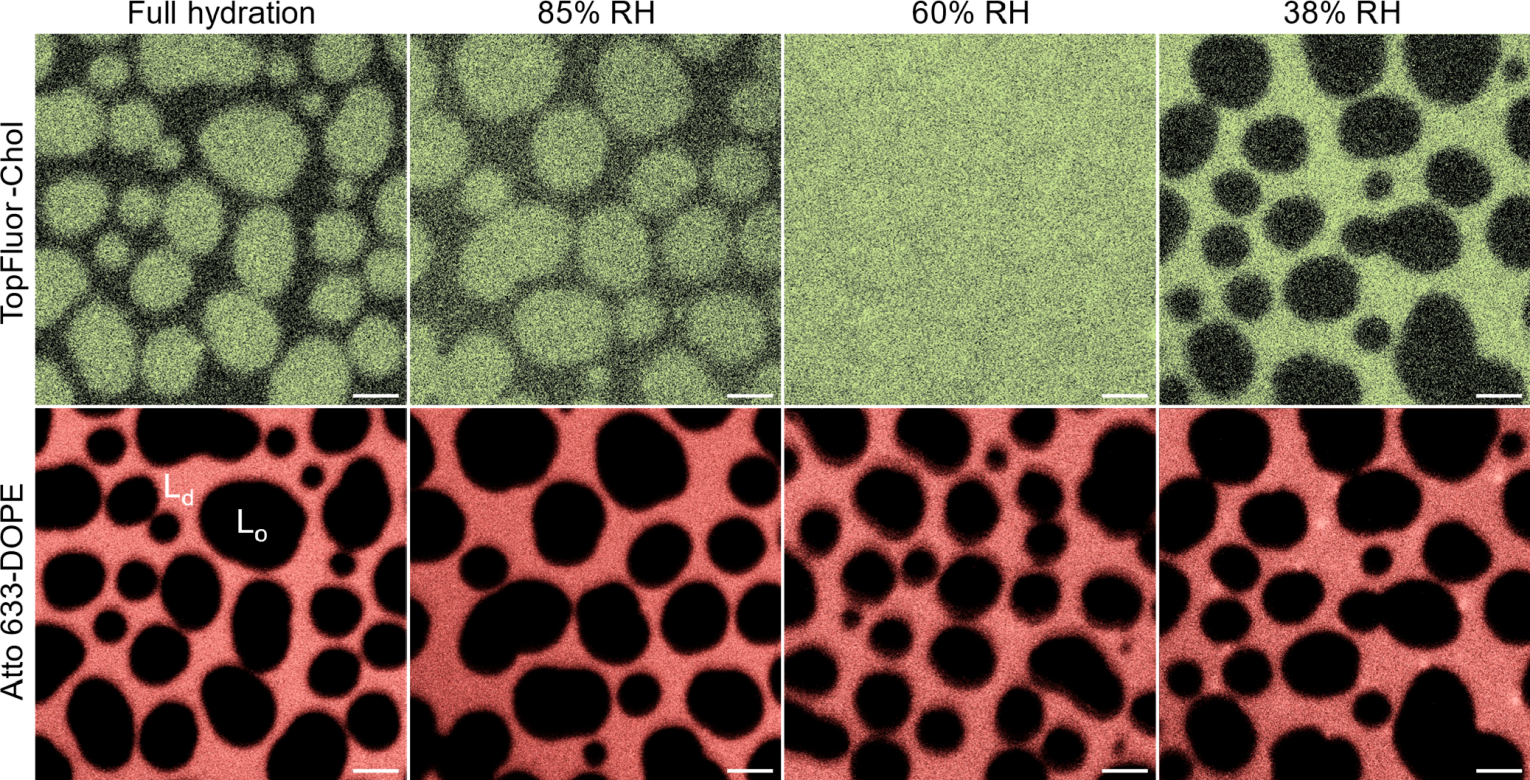
Exemplary confocal fluorescence microscopy images of a double-labeled, phase-separated SLB composed of an equimolar mixture of 14:1 PC, Chol, and SM at four membrane hydration states. Chol, which partitions in both the L_o_ and L_d_ phases, is labeled with the TopFluor probe (green, upper row), while the L_d_ phase is labeled with Atto 633-DOPE (pink, bottom row). Images for each hydration state originate from distinct sample areas. The concentration of each fluorescent probe was 0.1 mol %. The scale bar corresponds to 2.5 μm.

As shown in Figure 1, under fully hydrated conditions, TopFluor-Chol preferentially localizes in SM and Chol-rich L_o_ domains, evident from the higher fluorescence intensity in L_o_ compared to L_d_ regions. This observation aligns with previous findings for hydrated giant unilamellar vesicles (GUVs) and cell-derived giant plasma membrane vesicles (GPMVs), which exhibited analogous phase separation.^23^ However, we note that as membrane hydration decreases, the contrast diminishes, then completely disappears, and eventually reverses. In contrast, the distribution of Atto 633-DOPE remains constant regardless of the membrane hydration state, maintaining a strong preference for the L_d_ phase. Consequently, the overall membrane structure remains unaltered during dehydration (and rehydration) of the phase separated membrane. Neither does phase separation disappear, nor does phase inversion occur at any membrane hydration state. Our previous research provides extensive information on this matter.^24^

To quantify the hydration-dependent distribution of TopFluor-Chol between distinct phases, we extracted fluorescence intensities (*I*_*Lo*_ and *I*_*Ld*_) of the respective phases, using the green channel of the confocal images. Given that previous findings demonstrated the fluorescence quantum yield of TopFluor-Chol to be virtually independent of the lipid environment, specifically in the L_o_ and L_d_ phases,^23^ we assumed that the fluorescence intensity is proportional to the relative concentration of fluorescently labeled cholesterol in the membrane phases. Consequently, to calculate the partitioning coefficient of TopFluor-Chol in the L_o_ and L_d_ phases, we employed the following equations: *xL_o_* = *I_Lo_*/(*I_Lo_* + *I_Ld_*), *xL_d_* = *I_Ld_*/(*I_Lo_* + *I_Ld_*), respectively. According to this definition, 0.5 < *xL*_*o*_ ≤ 1 implies that the affinity of TopFluor-Chol for the L_o_ phase is stronger than for the L_d_, whereas a value in the range 0 ≤ *xL_o_* < 0.5 means that TopFluor-Chol favors the L_d_ phase over the L_o_ environment. *xL_o_* equal to 0.5 is indicative of no lipid phase-selectivity. The values of *xL_d_* should be interpreted analogously. The resulting partitioning coefficients *xL_o_* and *xL_d_* as a function of membrane hydration state are depicted in Figure 2.

**Figure 2:**
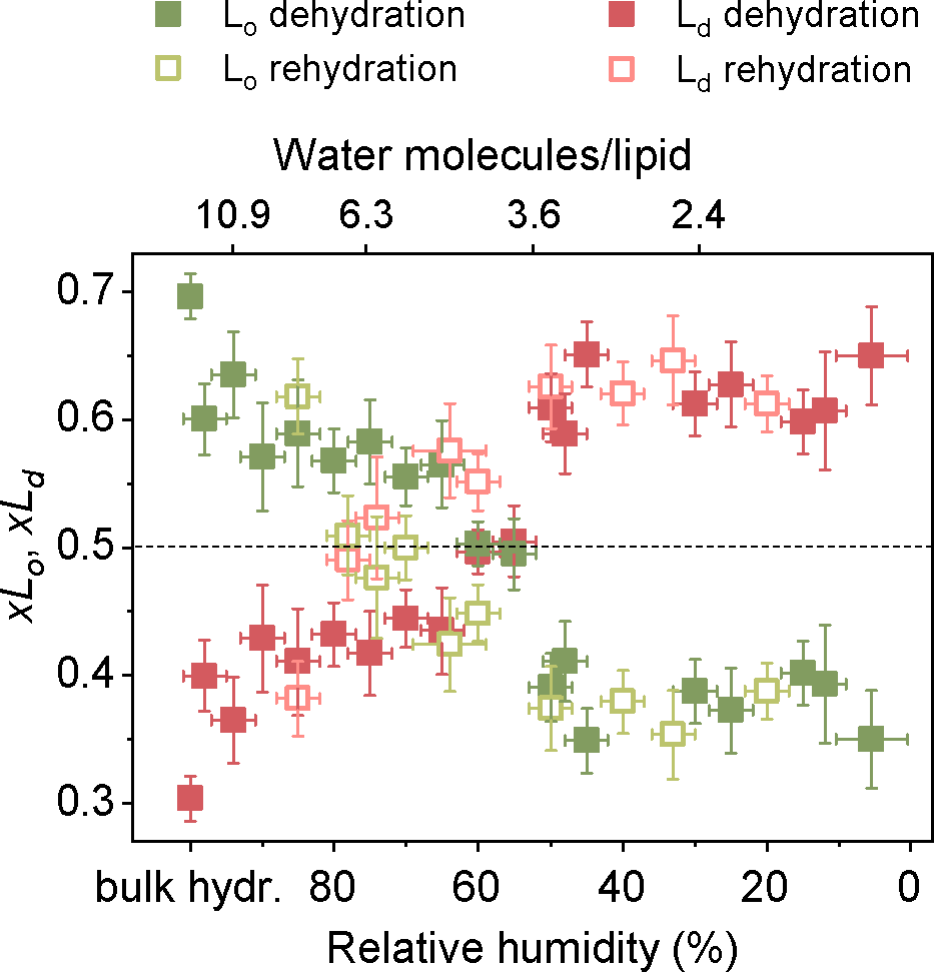
Partitioning coefficients (*xL_o_*, *xL_d_*) of TopFluor-Chol in the L_o_ and L_d_ phases within the 14:1 PC:SM:Chol (1:1:1) SLBs equilibrated in atmosphere of different relative humidity levels during dehydration (solid squares) and rehydration (open squares). Each data point represents the partitioning coefficient calculated based on the average fluorescence intensities from at least 6 spots of distinct phase from each sample at a specific membrane hydration state. The number of samples varies with membrane hydration state, ranging from 1 to 3.

As shown in Figure 2, under bulk membrane hydration, the partitioning coefficient of TopFluor-Chol in L_o_ domains equals to 0.70 ± 0.02, and correspondingly 0.30 ± 0.02 in the L_d_ phase, indicating its preference for an SM-rich environment. We note here that the partitioning coefficient of TopFluor-Chol for the L_o_ phase has been reported to be equal to 0.80 ± 0.03 in GUVs and 0.66 ± 0.60 in cell-derived GPMVs.^23^ Importantly, the values we report here are in full quantitative agreement with the partitioning coefficient of native cholesterol between coexisting L_o_ and L_d_ phases (∼0.7 and ∼0.3, respectively) in fully hydrated SLBs of the same composition.^25^ Consequently, it can be inferred that, under these specific conditions, TopFluor-Chol accurately mimics native cholesterol in terms of its lateral distribution in the phase-separated lipid bilayer.

Surprisingly, upon removal of bulk water and equilibration of the membrane with an atmosphere of high relative humidity (∼95% RH), the *xL_o_* drops to a value slightly above 0.6. Then, it gradually decreases with further reduction in water content, ultimately reaching a plateau with an average value of 0.38 within the hydration range of 5–50% RH. Notably, this effect is reversible, as demonstrated during the rehydration of the SLB (see Figure 2).

To ascertain whether the lateral distribution of TopFluor-Chol accurately reflects the native behavior of Chol, not only under fully hydrated conditions but also under reduced hydration, we first had to rule out the possibility that Chol migration toward surrounding unsaturated lipids was solely due to the expulsion of the relatively bulky TopFluor moiety, aiming to escape tight SM-rich regions during dehydration. To address this concern, we repeated the dehydration/rehydration experiment, replacing TopFluor-Chol with analogously labeled SM (TopFluor-SM). The results of this experiment are demonstrated in the Supporting Information in Figure S1. In brief, the partitioning coefficients of labeled SM in the L_o_ and L_d_ phases remain unchanged regardless of the membrane hydration state. However, it is noteworthy that the SM tagged with TopFluor favors the L_d_ phase, with *xL_o_* and *xL*_*d*_ averaging 0.39 and 0.61, respectively. Despite this, there remains a considerable amount of TopFluor-SM molecules in the L_o_ domains, which could potentially diffuse into the surrounding L_d_ phase upon membrane dehydration. However, such migration does not occur.

To gain a detailed molecular level insight into the dehydration-driven cholesterol redistribution between lipid raft and nonraft environment, we employed atomistic molecular dynamics simulations of three kinds of systems: (i) fully hydrated SM:Chol (3:2) and 14:1 PC:Chol (3:1) bilayers to represent experimentally resolved, fully hydrated L_o_ and L_d_ phases with their respective cholesterol content, (ii) fully hydrated 14:1 PC:SM:Chol (1:1:1) membrane, initialized with randomly mixed lipid molecules to confirm that we can observe the onset of phase separation and Chol migration toward SM-rich regions, and (iii) gradually dehydrated 14:1 PC:SM:Chol (1:1:1) system down to hydration level corresponding to 8 water molecules per lipid, to check the reversal of Chol preference from SM to phosphatidylcholine (PC) lipids and to analyze membrane organization under low water availability. We note that, although lower hydration levels were achievable, lipid bilayer became increasingly unstable which manifested itself in isolated, random events (single occurrences per hundreds of ns of simulation time) of irreversible surface penetration by lipid hydrocarbon chains. Accordingly, we decided to limit dehydration at the level allowing for stable runs of desired length.

Our simulations of fully hydrated, initially randomly mixed 14:1 PC:SM:Chol bilayer indicated a gradual separation of 14:1 PC and SM into membrane patches (Figure S2), with Chol exhibiting the expected tendency to colocalize with SM rather than PC lipids as the simulation progressed (Figures 3 and S3, fully hydrated condition). This observation validates the force field used for simulations, and notably, it demonstrates that the experimentally observed partitioning of TopFluor-Chol between L_o_ and L_d_ phases is indeed driven by the Chol moiety. Remarkably, gradual dehydration of the system down to 8 water molecules per lipid led to the inversion of Chol preference from the SM to PC phase (Figures 3 and S3, partially dehydrated condition), again in agreement with experimental findings.

**Figure 3:**
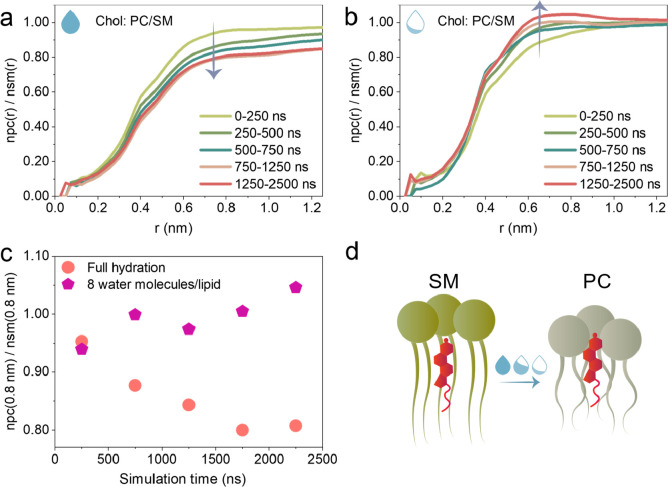
(a, b) Ratio of cumulative radial distribution functions of PC and SM lipids around cholesterol at different simulation times for lipid bilayer in fully hydrated and partially dehydrated (8 water molecules per lipid) conditions, respectively. (c) Evolution of PC/SM ratio within 0.8 nm distance around cholesterol over simulation time for 14:1 PC:SM:Chol lipid bilayer under fully hydrated and partially dehydrated conditions. (d) Cartoon representation of a cholesterol colocalization trend.

The transition from fully hydrated PC:Chol and SM:Chol phases to a perturbed hydration regime was accompanied by an increase in overall membrane thickness and hydrocarbon chain ordering, with the PC phase showing significantly larger effects compared to the already thick and ordered SM phase (Table S1). In either phase, the distribution of Chol across the membrane normal shifted outward from the midplane, following the trends observed for its neighboring lipid head distributions (Figure S4). In the case of Chol in the PC phase, however, the effect of dehydration was further augmented by an additional ∼0.1 nm displacement toward the bilayer surface, relative to the displacement of PC phosphate groups, yielding a 26% reduction in the distance between the Chol’s hydroxyl group and lipid’s phosphate moiety (Figure 4 and Table S1).

**Figure 4:**
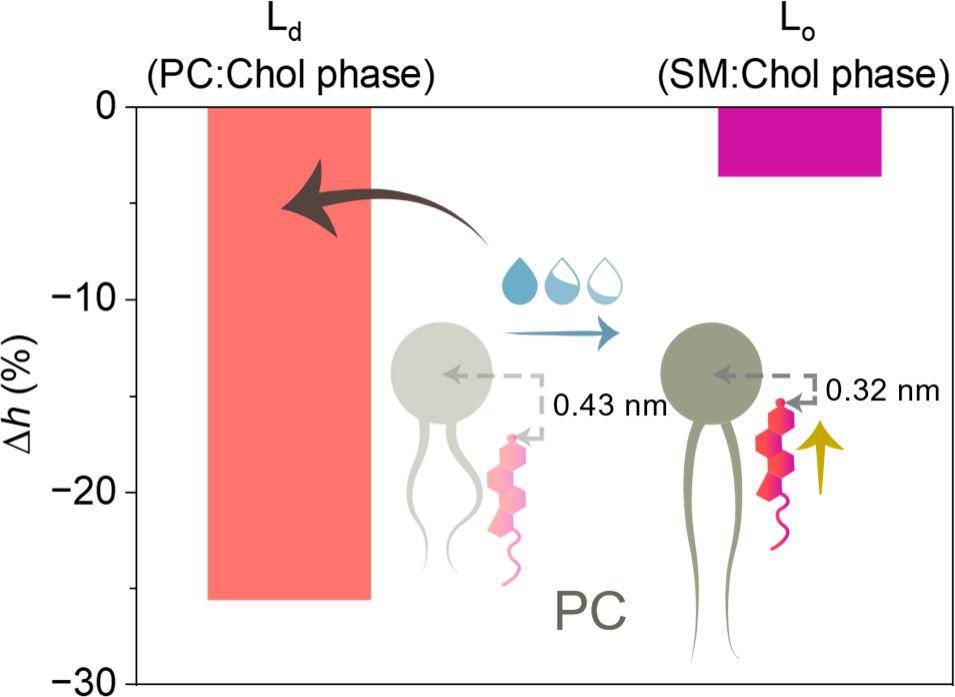
Percentage change in the distance (*h*) between the O atom in the hydroxyl group of Chol and the P atom in the phosphate of the lipid headgroup, PC or SM in the respective phase, upon dehydration down to 8 water molecules per lipid, calculated relative to fully hydrated conditions, along with a schematic representation of the observed scenario for Chol in the PC phase. The absolute values for SM can be found in Table S1.

The different response of PC and SM phases to dehydration was reflected by differences in hydrogen bond populations (Figure 5, Tables S2–S4). Overall, following limited availability of the aqueous solvent, PC lipids lost more interactions with water molecules than did SM lipids (Figure 5b). Intriguingly, while SM lipids lost similar fraction of hydrogen bonds within the interfacial region as in the headgroup region, PC lipids lost significantly more hydrogen bonds in the interfacial region compared to the headgroup region (35% vs 11%, respectively). Chol molecules, which were already better hydrated in the PC-rich compared to SM-rich phase under full solvent availability (1.2 vs 1.1 H-bonds with water per Chol molecule, respectively), were found to preserve their interaction with water more efficiently among PC than SM lipids (loss of 9% vs 22% lipid–water hydrogen bonds per Chol molecule, respectively, Figure 5c). In both phases, the reduction of Chol interaction with water was compensated by an increase of its interaction with neighboring lipids (Table S4). Notably, however, in the case of PC-rich phase additional hydrogen bonds were formed with PC headgroups, whereas in the SM-rich phase, new interactions were created within the interfacial region (Figure 5d).

**Figure 5:**
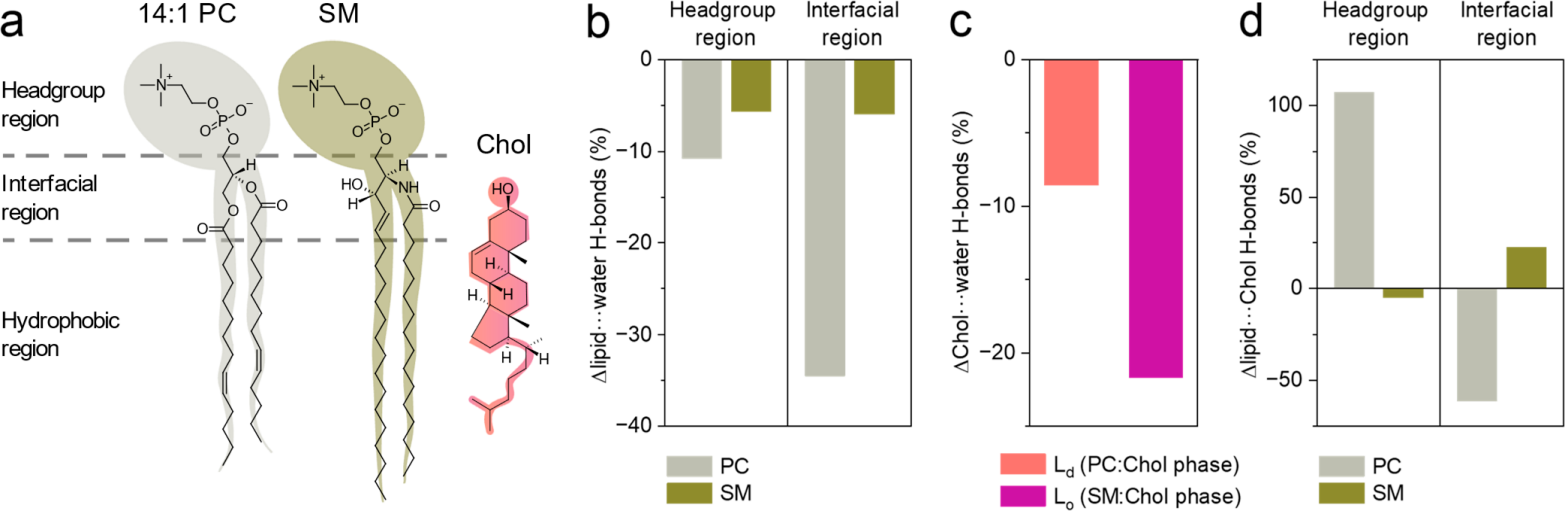
(a) Molecular structures of the studied lipids: 14:1 PC, SM and Chol. Percentage change in the number of hydrogen bonds (b) between lipids (PC and SM) and water (per lipid), (c) between Chol and water (per Chol), and (d) between Chol’s hydroxyl and lipids (per Chol). Panels (b)–(d) depict the effects observed upon dehydration down to 8 water molecules per lipid and are calculated relative to fully hydrated conditions. See the Supporting Information for the absolute values (Tables S2–S4).

The compiled experimental and computational evidence indicates that, under conditions of low membrane hydration, cholesterol exhibits a stronger affinity for unsaturated phosphatidylcholine-rich over sphingomyelin-rich regions. Moreover, the redistribution of cholesterol between coexisting domains induced by dehydration is not specific to the TopFluor-Chol probe but does occur with native, untagged Chol as well. However, a question remains: What is the driving force behind this peculiar behavior of cholesterol, unprecedented in previous observations?

Numerous studies have established that cholesterol tends to favor phospholipids featuring saturated acyl chains, as opposed to those with unsaturated acyl chains.^26−28^ Nevertheless, it is clear that factors beyond acyl chain saturation also play a significant role in cholesterol-phospholipid interactions. This is exemplified by the observation that under fully hydrated conditions cholesterol prefers interacting with SM over PC lipids, even at equal acyl chain order (and hydrophobic length).^29^ While SM and PC share the same headgroup, they differ in the headgroup-tails linkage. Unlike the glycerol linkage present in PC lipids, the presence of a sphingosine linkage imparts SM with not only hydrogen bond acceptor groups but also with donor ones (NH and OH). These hydrogen bonding capabilities, along with acyl chain order and hydrophobic length, appear to promote the preferential association of cholesterol with sphingomyelin under full hydration.

Under fully hydrated conditions, due to a higher chain order and more carbons in the hydrophobic chains of sphingomyelin compared to unsaturated phosphatidylcholine molecules, the L_o_ domains exhibit greater thickness compared to the surrounding continuous L_d_ phase. Upon membrane dehydration, this height disparity at the interface of coexisting phases (hydrophobic mismatch) decreases linearly.^30^ Our previous atomic force microscopy study revealed that under mild dehydration conditions (90% RH, ca. 10 water molecules/lipid), a thickness of the L_d_ phase equals to 3.87 nm, while the height mismatch was 1.36 nm, yielding a thickness of the L_o_ phase of 5.23 nm.^30^ The hydrophobic mismatch between the raft-like and nonraft phases decreased nearly 2-fold (from 1.36 to 0.8 nm) as membrane hydration decreased from approximately 10 to less than 1 water molecule per lipid.^30^ The results of MD simulations presented herein indicate that the decrease in hydrophobic mismatch primarily stem from the dehydration-induced thickening of the L_d_ phase, resulting from the ordering of PC lipid acyl chains.

As such, the differences in lipid phase characteristics, such as thickness and ordering, become less pronounced under low solvent availability. We propose that this fosters more efficient penetration of the PC compartment by Chol. An additional driving force for Chol redistribution seems to arise from the need to satisfy lipid hydrogen bonds, particularly within highly polar and solvent-exposed phosphate groups, which become increasingly difficult to maintain with lowering water content. In this regard, PC molecules, which lose more interaction with water than SM lipids upon dehydration (likely due to the absence of H-bond donor groups in contrast to SM), offer more opportunities to interact with Chol’s hydroxyl group, especially given the dehydration-induced upward shift toward the membrane surface.

The cholesterol influx from the L_o_ domains into surrounding unsaturated lipid-rich regions rationalizes well the phenomenon observed in our previous experimental study using the environment-sensitive fluorescent probe Laurdan.^25^ In brief, we compared changes of the L_d_ phase’s fluidity, determined from the Laurdan emission spectrum, as a function of the hydration state of two types of SLBs: (i) a membrane with coexisting L_d_ and L_o_ domains, identical in composition to the current study, but with only Atto 633-DOPE and Laurdan as fluorescent labels (so Chol was native, untagged), and (ii) a single-phase membrane with the same molecular composition as the L_d_ phase in the phase-separated membrane (containing 0.3 mol fraction of Chol). While the two systems were compositionally identical under fully hydrated conditions, dehydration yielded disparate results. A decrease in the fluidity of the L_d_ phase upon dehydration was observed in both systems, however, the changes in the phase-separated membrane were smaller (approximately by a factor of 2 at low hydrations). This pointed toward an additional mechanism that counteracts and moderates the effects of L_d_ dehydration in the phase-separated membrane. In light of our current findings, we can now unambiguously state that in the membrane with L_o_ domains, as dehydration progresses, Chol migrates from the L_o_ domains to the surrounding unsaturated lipid-rich L_d_ regions, preventing excessive stiffening of the membrane. In other words, Chol fluidizes a dehydrated L_d_ environment.

We note that our findings have important implications for the membrane fusion process. The hydrophobic mismatch between the L_d_ and L_o_ domains gives rise to an interfacial force known as line tension at the boundary between the two phases. In prior study, we found that reduction in hydration from approximately 10 to less than 1 water molecule per lipid, in the same membrane system as studied herein, governed a 3.5-fold decline in line tension (from approximately 7 to 2 pN).^30^ Cholesterol, in general, can either increase or decrease the thickness of a phospholipid bilayer, depending on the lipid chain length, saturation, and lipid phase. However, it has been established that for lipids containing 12–16 carbons per chain, cholesterol thickens the bilayer regardless of saturation and phase.^31,32^ Therefore, in our case, where L_d_ lipids have 14 carbons and L_o_ lipids have 16 carbons per chain, we assume that Chol increases the thickness of both 14:1 PC lipids in the L_d_ phase and SM lipids in the L_o_ phase. Consequently, the depletion of Chol in L_o_ domains and the associated enrichment of Chol in the surrounding L_d_ phase lead to a decrease in hydrophobic mismatch. Therefore, both the dehydration and the redistribution of cholesterol between domains contribute to a decrease in hydrophobic mismatch and the resulting reduction in line tension, minimizing the boundary energy of the L_o_ domains.

To estimate the possible energy gain associated with a reduction in line tension, we employ a simplistic model describing the boundary energy of an individual raft-like domain surrounded by a continuous fluid phase as *E* = γ*L*, where γ represents the line tension, and *L* denotes the domain perimeter.^15^ In our investigations, we did not observe notable alterations in the perimeter of the L_o_ domains during dehydration, leading as to assume that it is constant, with changes occurring only in line tension. Based on our previous atomic force microscopy studies, a decrease in membrane hydration from approximately 10 to fewer than 1 water molecule per lipid resulted in a reduction in line tension by around 5 pN.^30^ Addressing a more realistic size for the raft in the cell membrane, we consider a raft with a diameter of 20 nm in calculations. Under these assumptions, the energy gain is estimated to be on the order of 70*k*_B_*T* (detailed calculations are provided in the Supporting Information), which falls within the range of free energy barrier predicted in theoretical models for membrane fusion.^33,34^ Therefore, it seems feasible that as the two membranes get closer and the dehydration of lipid headgroups progresses, more cholesterol is released from raft domains into the continuous more fluid phase. This, in tandem with a dehydration-induced ordering of lipid acyl chains, leads to a significant reduction in line tension at the distinct environment boundary. Consequently, this reduction produces an energy gain, facilitating the formation of the subsequent stalk intermediate. Furthermore, cholesterol counteracts the dehydration-induced extensive changes in fluidity of the nonraft membrane regions,^25^ thus creating a more favorable environment for the fusion.

In summary, we used confocal fluorescence microscopy and atomistic MD simulations to unravel cholesterol-phospholipid–water interactions and the organization of cholesterol in lipid bilayer with coexisting L_o_ and L_d_ phases under reduced hydration—a scenario reminiscent of temporal membrane dehydration during cellular events such as membrane fusion. We unveiled that dehydration of a biomimetic lipid bilayer drives cholesterol release from raft-like L_o_ domains into the surrounding L_d_ phase. The inversion of cholesterol preference from raft-like SM-rich domains to the surrounding unsaturated PC-rich phase underscores the regulatory role of water in shaping cell membrane structure.

Our MD simulation results suggest that a molecular rationale for dehydration-induced cholesterol redistribution between lateral domains, involves two primary coacting factors. First, unsaturated PC lipids in the L_d_ phase exhibit greater susceptibility to structural changes upon dehydration compared to SM lipids in the L_o_ phase. As the membrane gets dehydrated, the acyl chains of the PC lipids undergo significant ordering and a concomitant increase in membrane thickness. Consequently, the biophysical properties of the two phases become more similar, facilitating the interaction of cholesterol with PC lipids. Second, upon membrane dehydration, unsaturated PC lipids lose more hydrogen bonds with water than SM molecules. In response to the excessive loss of hydrogen bonds by PC headgroups, cholesterol migrates toward the PC phase and shifts to the surface to form hydrogen bonds between its hydroxyl group and the phosphate moieties of the PC headgroups.

The dehydration-induced cholesterol release from lipid rafts into the surrounding phase may potentially play a role during cellular events involving membrane fusion. First, it maximizes the reduction of hydrophobic mismatch between domains of distinct phases (and thus the line tension), facilitating the energy release required for stalk formation. Second, lipid rafts likely serve as cholesterol reservoirs, releasing the sterol to regulate membrane fluidity. We emphasize the universality of the latter role, as continuous adjustments to membrane fluidity are necessary to ensure the integrity and functionality of biological membranes under diverse physiological conditions, extending beyond the context of membrane fusion.

## Supporting Information Available

Materials and methods; partitioning coefficient of TopFluor-SM in the L_o_ and L_d_ phases within the 14:1 PC:SM:Chol (1:1:1) SLB as a function of membrane hydration; evidence of phase separation under fully hydrated conditions from MD simulations; radial lipid distribution functions around cholesterol under fully hydrated and partially dehydrated conditions; number density profiles of water O atom, PC and SM P atoms, Chol O atom along Z axis in the respective phases under fully hydrated conditions and partially dehydrated conditions; structural parameters of simulated lipid bilayers under fully hydrated and partially dehydrated conditions; number of hydrogen bonds between lipids (including cholesterol) and water and between cholesterol and lipids under fully hydrated and partially dehydrated conditions; calculation of the energy released due to reduction of line tension (PDF)
