# Supplementary material for: Dehydration of Lipid Membranes Drives Redistribution of Cholesterol Between Lateral Domains
Source: J Phys Chem Lett. 2024 Apr 18;15(16):4515–22. doi: 10.1021/acs.jpclett.4c00332 (PMC11056968; doi:10.1021/acs.jpclett.4c00332)
Supplement: Supplementary file 1 — jz4c00332_si_001.pdf [file jz4c00332_si_001.pdf]

Supporting Information for

Dehydration of Lipid Membrane Drives Redistribution of Cholesterol Between Lateral Domains

Hanna Orlikowska-Rzeznik,[1*] Emilia Krok,[1] Maria Domanska,[2] Piotr Setny,[2] Anna Lągowska,[1] Madhurima Chattopadhyay,[1†] and Lukasz Piatkowski[1*]

[1]Faculty of Materials Engineering and Technical Physics, Poznan University of Technology, 60-965 Poznan, Poland

[2]Biomolecular Modelling Group, The Centre of New Technologies, University of Warsaw, 02-097 Warsaw, Poland

Correspondence: hanna.orlikowska@put.poznan.pl (H. Orlikowska-Rzeznik); lukasz.j.piatkowski@put.poznan.pl (L. Piatkowski)

Table of Content:

Materials and Methods

Supplementary Figures S1–S4

Supplementary Tables S1–S4

Calculation of the Energy Released Due to Reduction of Line Tension

Materials and Methods

Materials. Lipids: 1,2-dimyristoleoyl-glycero-3-phosphocholine (14:1 PC), egg yolk sphingomyelin (SM), cholesterol (Chol), and fluorescently labelled lipids: 23-(dipyrrometheneborondifluoride)-24-norcholesterol (TopFluor-Chol) and N-[11-(dipyrrometheneboron difluoride)undecanoyl]-D-*erythro*-sphingosylphosphorylcholine (TopFluor-SM) were provided by Avanti Polar Lipids (Alabaster, AL). Lipid 1,2-dioleoyl-sn-glycero-3-phosphoethanolamine labeled with Atto 633 (Atto 633-DOPE) and HPLC grade chloroform were acquired from Merck KGaA (Darmstadt, Germany). Buffer reagent 4-(2-hydroxyethyl)piperazine-1-ethanesulfonic acid (HEPES PUFFERAN) was purchased from Carl Roth GmbH + Co., KG (Karlsruhe, Germany). Calcium chloride ($CaCl_2$) was sourced from Chempur (Piekary Slaskie, Poland) and sodium chloride (NaCl) was supplied by PPH STANLAB Sp. z o.o. (Lublin, Poland). These compounds were used as purchased without further purification. Ultrapure water of 18.2 MΩ×cm resistivity was obtained using the Milli-Q Direct Water Purification System from Merck KGaA (Darmstadt, Germany). Optical adhesive UV-activated glue Norland 68 was purchased from Thorlabs Sweden AB (Mölndal, Sweden). Sheets of mica, which were used to prepare solid supports for the lipid bilayers, were obtained from Shree GR Exports Private Limited (Kolkata, India).

Solid-Supported Lipid Bilayers Fabrication. Solid-supported lipid bilayers (SLBs) were prepared using a vesicle deposition on a solid substrate method, as described elsewhere.[1–3] We investigated SLBs composed of an equimolar ternary mixture 14:1 PC:Chol:SM with 0.1 mol% of Atto 633-DOPE and 0.1 mol% of additional fluorescent label, either TopFluor-Chol or TopFluor-SM. The membrane components were mixed in chloroform with a final lipid concentration of 10 mM. Subsequently, the relevant solution was dried using nitrogen gas and then subjected to desiccation in a vacuum chamber for a minimum of 2 hours. The lipid film was hydrated using a buffer solution (containing 10 mM HEPES and 150 mM NaCl, pH adjusted to 7.4) to achieve a lipid concentration of 10 mM. The lipid suspension underwent four cycles of heating to 60°C and vortexing, with each heating and vortexing step lasting 1 minute. The lipid mixture was diluted tenfold in a buffer to obtain a 1 mM vesicle suspension, and then distributed into glass vials for storage at -20°C for subsequent use. A portion of the MLV suspension, with the desired composition, was bath-sonicated for a minimum of 10 minutes. To prepare a solid support for the deposition of vesicles, a thin sheet of freshly cleaved mica, pre-cut into round plates with a 9 mm diameter, was fixed on a glass coverslip with UV-activated glue. Subsequently, a plastic cylinder, created by removing the lid and bottom of a microcentrifuge tube was placed on mica and sealed with silicone to create a reservoir with mica at the bottom. Next, 100 µL of 1 mM vesicle suspension was deposited onto the mica surface, followed by the immediate addition of 2 µL of a 0.1 M $CaCl_2$ solution. After ca. 30 seconds, 600 µL of the previously mentioned buffer solution was introduced to prevent the dehydration of the membrane under formation. Following a 30-minute incubation at room temperature, the supported lipid bilayer was rinsed with 20 mL of buffer solution to remove excess unburst vesicles. Ultimately, pure buffer solution was used to fill the remaining space in the reservoir, a condition referred to as bulk hydration throughout the paper.

SLB Hydration-State Control. To control the lipid membrane hydration state, we utilized our custom-designed humidity control system, described previously.[1–3] The setup includes several components: a nitrogen gas (N_2) cylinder, three flow meters, three manual valves, a water reservoir (for achieving water vapor saturation), and an electronic hygrometer. Initially, to decrease the hydration of the SLB, bulk water was extracted from the sample container using a micropipette until no buffer droplets were visible on the mica surface. Subsequently, nitrogen gas with a relative humidity of 95% RH was gently introduced into the sample container. The RH of the nitrogen gas was controlled by blending streams of wet (water-vapor-saturated, 95% RH) and dry (0% RH) gases. The individual flows of wet and dry N_2 gases were regulated using two manual valves, and the flow rates were monitored with two flow meters connected to their respective paths. To maintain a constant N_2 gas flow rate of approximately 1.2 L/min throughout the experiment, a third flow meter and manual valve were employed. An electronic hygrometer allowed continuous monitoring of both the relative humidity and temperature of the final gas stream, prompting adjustments as necessary. The de(re)hydration process was carried out gradually. After allowing approximately 10 minutes for the SLB atmosphere to equilibrate to the specified relative humidity, the sample was imaged.

Fluorescence Imaging. Confocal fluorescence imaging was performed on a Carl Zeiss LSM 710 laser-scanning confocal microscope, using 488 nm and 633 nm laser lines. The samples were illuminated and fluorescence was collected through a Carl Zeiss EC Plan-Neofluar 40x/1.30 oil immersion objective. The laser power was adjusted during imaging to prevent the dyes from undergoing excessive photobleaching.

Molecular Dynamics Simulations. Simulations represented symmetric bilayers of ~81 nm^2 area (9 nm × 9 nm) in the XY plane, composed of lipid types used in the experiments, embedded in aqueous environment. The following systems were constructed using the CHARMM-Gui[4] server: SM:Chol (90:60 lipids per monolayer), 14:1 PC:Chol (90:30 lipids per monolayer), 14:1 PC:SM:Chol (62:62:62 lipids per monolayer), each with 2.5 nm margin of water on both membrane sides. The systems were simulated at temperature T = 298 K, and ambient pressure, under periodic boundary conditions with default equilibration protocol provided by CHARMM-Gui and production runs of 0.5, 0.5 and 2.5 µs, respectively. After the first 0.3 µs of the simulation time, the snapshot of 14:1 PC:SM:Chol system was used to generate dehydrated bilayer: water molecules that were furthest away from the membrane were removed to leave ~13 water molecules per lipid. The system size in Z direction, perpendicular to the membrane plane, was increased from ~9 to 12 nm, in order to create ~7 nm thick water vapor layer. Following this modification, the system size in Z direction was fixed and semiisotropic barostat was applied only in X and Y directions. Effective simulation pressure in XY plane of −100 bar was adjusted by trial and error to achieve membrane area possibly close to that of the fully hydrated system (note that system cross-section along the Z axis contains lipid/liquid water phase and water vapor). After the system was equilibrated for 0.1 µs, the second, analogous dehydration stage, albeit with no further modification of the simulation box dimensions, was performed to achieve hydration level of 8 water molecules per lipid. The effective pressure was established at −30 bar and the system was simulated for 2.5 µs. All simulations were carried out with Gromacs software.[5] Charmm36 force field was used for lipids,[6] and TIP3P model was used for water, [7] with default simulation parameters for the force field used, as implemented in CHARMM-Gui input generator.

In order to determine the presence of PC and SM rich phases in the system, membrane plane was divided into 3 nm × 3 nm grid and lipid molar fractions were evaluated within each cell, separately for each leaflet. To increase sampling, the calculations were repeated with grid origin translated along 5×5 sub-grid spanning the reference cell of the 3 nm × 3 nm grid. The analysis of fully hydrated L_d and L_o phases was carried out based on their dedicated simulations, whereas the analysis of dehydrated PC:Chol and SM:Chol phases was conducted over grid areas with PC fraction among PC and SM lipids >0.9 and <0.1, respectively. Radial distribution functions were calculated between hydroxyl oxygen atoms of cholesterol and phosphorus atoms of PC and SM lipids. Lipid density profiles across the membrane, and radial distribution functions were calculated using standard Gromacs tools, hydrogen bonding and other analyses were conducted using MDAnalysis python package.[8]

Lipid Order Parameter Determination. The ordering of the lipid acyl chains was determined by calculation of the order parameter (S_{CH}). S_{CH} is a measure of the relative orientation of the carbon-hydrogen bonds with respect to the bilayer normal. It was calculated according to the formula:

$$S_{CH} = \frac{1}{2}\langle 3\,cos^2\,\theta - 1\rangle, \tag{1}$$

in which θ is the angle between the bilayer normal and the vector joining carbon atom to its hydrogen atom, and $\langle\ \rangle$ represents an ensemble average. Calculation of the order parameter of lipid acyl chains was carried out with g_lomepro software.[9]

Supplementary Figures S1–S4

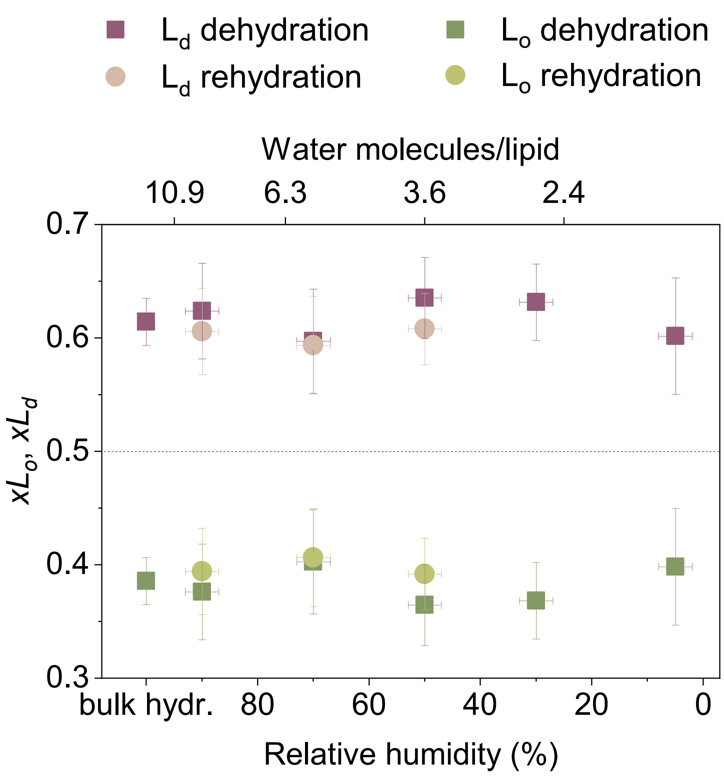

Figure S1. The partitioning coefficients of TopFluor-SM in the liquid-ordered (L_o) and liquid-disordered (L_d) phases within the 14:1 PC:SM:Chol (1:1:1) SLBs equilibrated in atmosphere of different relative humidity levels during both dehydration (squares, darker colours) and rehydration (circles, lighter colours). Each data point represents the partitioning coefficients calculated based on the average fluorescence intensities from at least 20 spots of distinct phase at a specific membrane hydration state from each of the two samples.

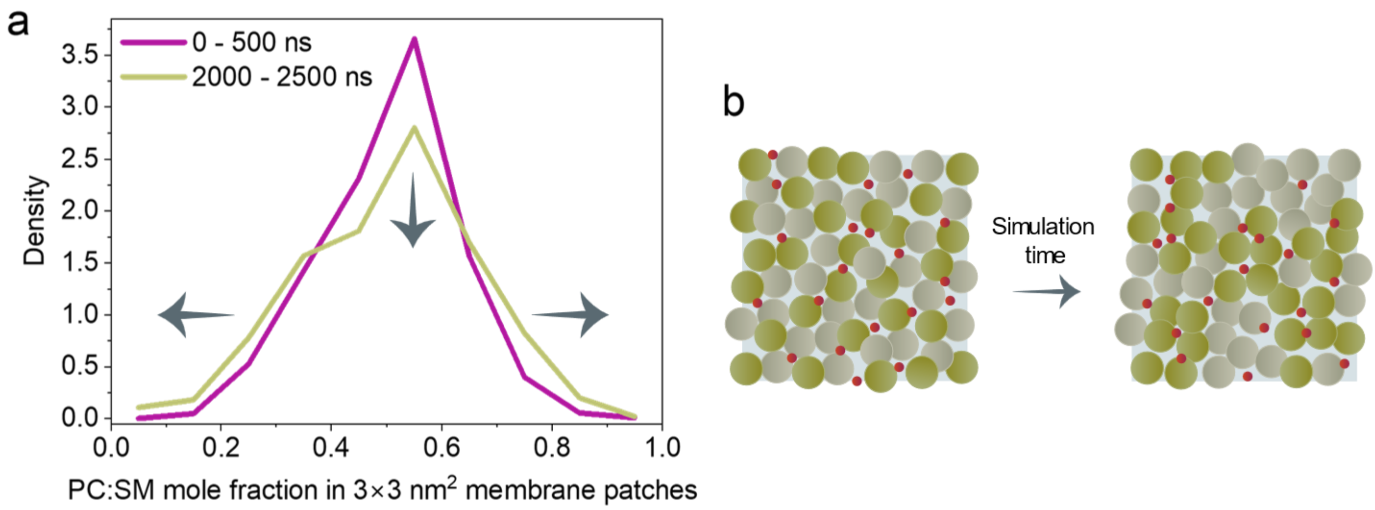

Figure S2. (a) The distribution of PC:SM mole fractions in 3×3 nm² membrane patches in the simulated 14:1 PC:SM:Chol (1:1:1) lipid bilayer under bulk hydration. (b) The cartoon representation of the phase separation trend as the simulation progressed.

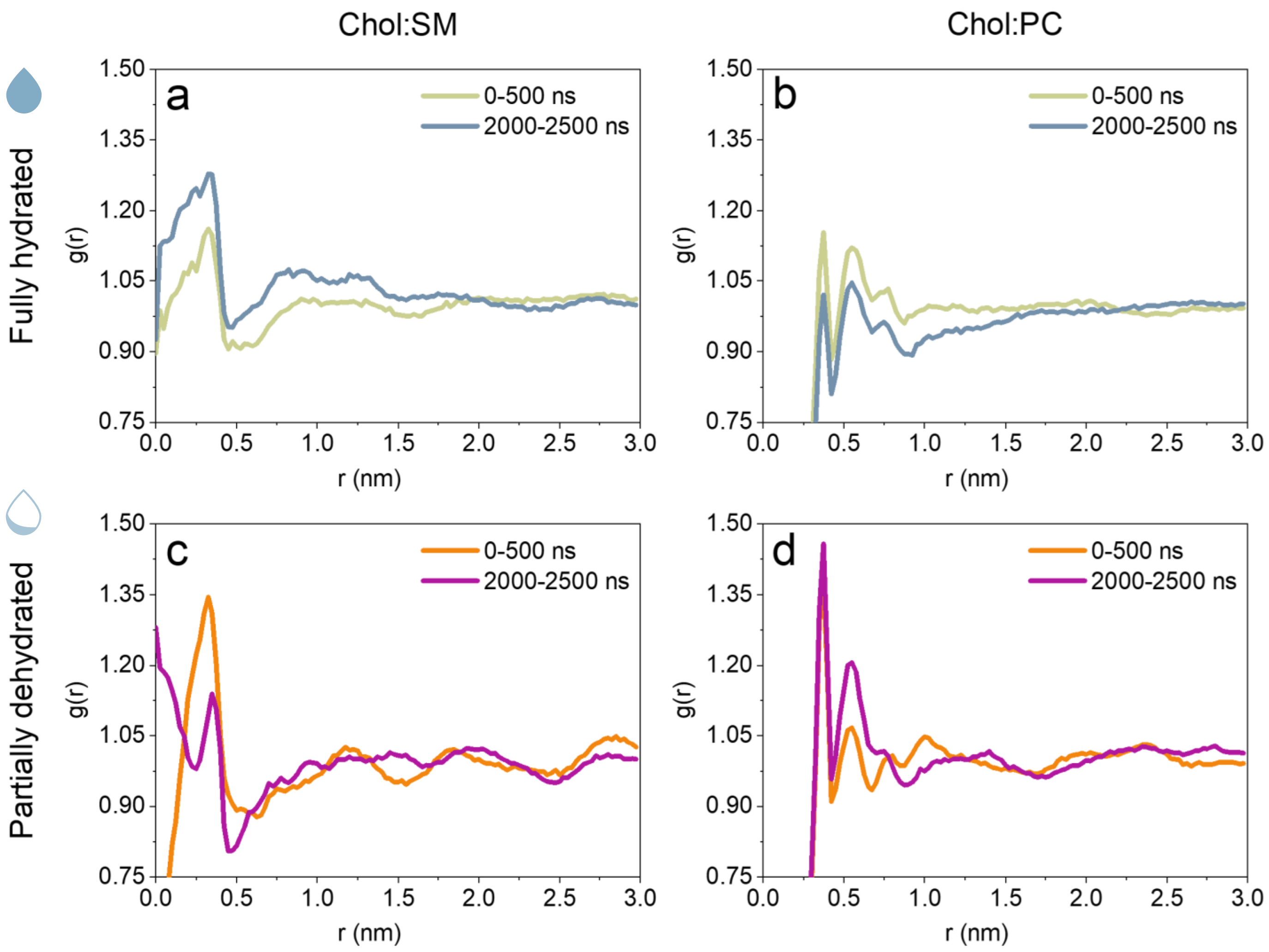

Figure S3. Radial distribution functions of 14:1 PC and SM lipids around cholesterol at the beginning (0–500 ns) and at the end (2000–2500 ns) of 14:1 PC:SM:Chol (1:1:1) lipid bilayer simulations under (a,b) fully hydrated and (c,d) partially dehydrated conditions (8 water molecules per lipid). Note that non-zero g(r=0) values for Chol:SM pair result from a tendency of Chol hydroxyl groups to remain covered under SM head groups.

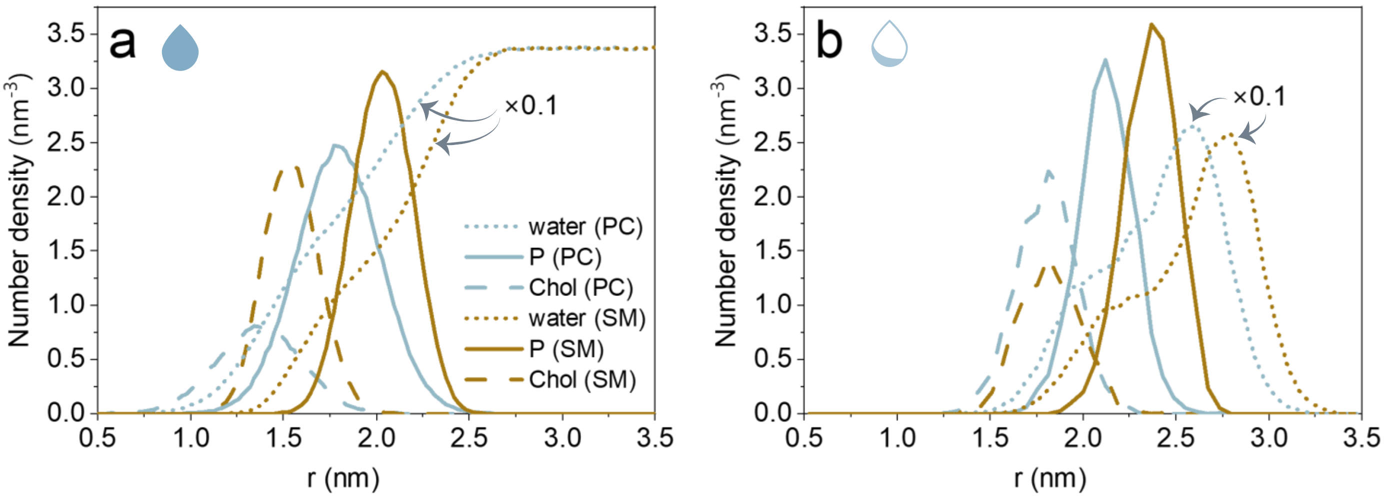

Figure S4. Number density profiles of water O atoms, PC and SM P atoms, Chol O atoms along Z axis in the respective phases in (a) fully hydrated conditions and (b) partially dehydrated conditions (8 water molecules per lipid).

Supplementary Tables S1–S4

Table S1. Structural parameters of simulated lipid bilayers under different hydration conditions (full hydration and partial dehydration down to 8 water molecules per lipid): membrane thickness (*d*), defined as averaged lipid P-P atom distance, cholesterol O atom position along bilayer normal with respect to lipid P atom position (*h*), and an average order parameter of the lipid acyl chains (S_{CH}).

Membrane hydration	*d* (nm)		*h* (nm)		S_{CH}	
	L_d (PC:Chol phase)	L_o (SM:Chol phase)	PC	SM	PC	SM
Full hydration	3.59	4.65	-0.43	-0.56	0.156	0.374
Partial dehydration	4.25	4.72	-0.32	-0.54	0.283	0.369

Table S2. Number of hydrogen bonds with water per lipid under different hydration conditions: full hydration and partial dehydration down to 8 water molecules per lipid. The headgroup region includes the oxygens of the phosphate group (PO_4) in both lipids, while the interfacial region involves oxygens of the carbonyl groups (CO) in PC, and oxygen and nitrogen atoms of the amide group (CO, NH) and oxygen atom of the hydroxyl group in SM.

Membrane hydration	PC···water			SM···water		
	Headgroup region	Interfacial region	In total	Headgroup region	Interfacial region	In total
Full hydration	4.33	1.24	5.56	3.89	1.55	5.44
Partial dehydration	3.86	0.81	4.67	3.67	1.46	5.13

Table S3. Number of hydrogen bonds with water per cholesterol under different hydration conditions: full hydration and partial dehydration down to 8 water molecules per lipid.

Membrane hydration	Chol···water	
	L_d (PC:Chol phase)	L_o (SM:Chol phase)
Full hydration	1.18	1.06
Partial dehydration	1.08	0.83

Table S4. Number of hydrogen bonds between cholesterol and lipids per cholesterol molecule under different hydration conditions: full hydration and partial dehydration down to 8 water molecules per lipid. The headgroup region includes the oxygens of the phosphate group (PO_4) in both lipids, while the interfacial region involves oxygens of the carbonyl groups (CO) in PC, and oxygen and nitrogen atoms of the amide group (CO, NH) and oxygen atom of the hydroxyl group in SM.

Membrane hydration	Chol···PC			Chol···SM		
	Headgroup region	Interfacial region	In total	Headgroup region	Interfacial region	In total
Full hydration	0.107	0.113	0.220	0.058	0.287	0.345
Partial dehydration	0.222	0.044	0.266	0.055	0.351	0.407

Calculation of the Energy Released Due to Reduction of Line Tension

To estimate changes of the energy of the system upon a reduction in line tension, we employ a simplistic model describing the boundary energy of an individual raft-like domain surrounded by a continuous fluid phase as E = gL, where g represents the line tension, and L denotes the domain perimeter.[10] We assumed the L to be constant, as we did not observe notable alterations in the perimeter of the L_o domains during dehydration. Consequently, in our calculations, the g is the only parameter that changes. Based on our previous atomic force microscopy study, a decrease in membrane hydration from approximately 10 to fewer than 1 water molecule per lipid (from 90% to 5% relative humidity) resulted in a reduction in line tension from around 7 pN to 2 pN.[2] For estimation, we consider a raft with a diameter of 20 nm. Change in the boundary energy of an individual raft is then:

$$\Delta E = E_{deh} - E_{hyd} = (\gamma_{deh} - \gamma_{hyd})L\,, \tag{2}$$

where subscripts *deh* and *hyd* denotes low and high hydration conditions, respectively.

$$\Delta E = (2\,pN - 7\,pN) \cdot 2\pi \cdot 10\,nm = -5\,pN \cdot 20\pi\,nm = -100\pi\,pN \cdot nm \approx -314\,pN \cdot nm$$

A negative value of the energy change is indicative of the reduction of the boundary energy of the system associated with the free energy release.

The thermal energy at physiological temperature of 37°C is:

$$k_BT \approx 1.38 \cdot 10^{-23}\,J \cdot K^{-1} \cdot 310\,K = 427.8 \cdot 10^{-23} J \approx 4.3 \cdot 10^{-21}\,N \cdot m \approx 4.3\,pN \cdot nm$$

$$\left|\frac{\Delta E}{k_BT}\right| = \left|\frac{-314\,pN \cdot m}{4.3\,pN \cdot m}\right| \approx 73$$

Therefore, the possible free energy, released during dehydration-driven cholesterol redistribution between lipid raft and non-raft environment can be on the order of $70k_BT$.

References

(1) Orlikowska-Rzeznik, H.; Krok, E.; Chattopadhyay, M.; Lester, A.; Piatkowski, L. Laurdan Discerns Lipid Membrane Hydration and Cholesterol Content. *J. Phys. Chem. B* **2023**, *127*, 3382–3391.

(2) Krok, E.; Franquelim, H. G.; Chattopadhyay, M.; Orlikowska-Rzeznik, H.; Schwille, P.; Piatkowski, L. Nanoscale Structural Response of Biomimetic Cell Membranes to Controlled Dehydration. *Nanoscale* **2024**, *16*, 72–84.

(3) Chattopadhyay, M.; Krok, E.; Orlikowska, H.; Schwille, P.; Franquelim, H. G.; Piatkowski, L. Hydration Layer of Only a Few Molecules Controls Lipid Mobility in Biomimetic Membranes. *J. Am. Chem. Soc.* **2021**, *143*, 14551–14562.

(4) Jo, S.; Kim, T.; Iyer, V. G.; Im, W. CHARMM-GUI: A Web-Based Graphical User Interface for CHARMM. *J. Comput. Chem.* **2008**, *29*, 1859–1865.

(5) Abraham, M. J.; Murtola, T.; Schulz, R.; Páll, S.; Smith, J. C.; Hess, B.; Lindah, E. Gromacs: High Performance Molecular Simulations through Multi-Level Parallelism from Laptops to Supercomputers. *SoftwareX* **2015**, *1–2*, 19–25.

(6) Klauda, J. B.; Venable, R. M.; Freites, J. A.; O'Connor, J. W.; Tobias, D. J.; Mondragon-Ramirez, C.; Vorobyov, I.; MacKerell, A. D.; Pastor, R. W. Update of the CHARMM All-Atom Additive Force Field for Lipids: Validation on Six Lipid Types. *J. Phys. Chem. B* **2010**, *114*, 7830–7843.

(7) Jorgensen, W. L.; Chandrasekhar, J.; Madura, J. D.; Impey, R. W.; Klein, M. L. Comparison of Simple Potential Functions for Simulating Liquid Water. *J. Chem. Phys.* **1983**, *79*, 926–935.

(8) Michaud-Agrawal, N.; Denning, E. J.; Woolf, T. B.; Beckstein, O. MDAnalysis: A Toolkit for the Analysis of Molecular Dynamics Simulations. *J. Comput. Chem.* **2011**, *32*, 2319–2327.

(9) Gapsys, V.; De Groot, B. L.; Briones, R. Computational Analysis of Local Membrane Properties. *J. Comput. Aided. Mol. Des.* **2013**, *27*, 845–858.

(10) Yang, S. T.; Kiessling, V.; Tamm, L. K. Line Tension at Lipid Phase Boundaries as Driving Force for HIV Fusion Peptide-Mediated Fusion. *Nat. Commun.* **2016**, *7*, 11401.
